# Effects of Exercise Training on Left Ventricular Hypertrophy and Cardiac Regulation in Elite Athletes: Insight from a Composite Index Proxy of Autonomic Control

**DOI:** 10.3390/jcdd13070304

**Published:** 2026-07-02

**Authors:** Gianluigi Oggionni, Giuseppina Bernardelli, Mara Malacarne, Massimo Pagani, Antonio Spataro, Antonio Pelliccia, Daniela Lucini

**Affiliations:** 1 Department of Medical Biotechnology and Translational Medicine (Department of Excellence 2023–2027), University of Milan, 20129 Milan, Italy; gianluigi.oggionni@unimi.it (G.O.);; 2 Department of Clinical Sciences and Community Health (Department of Excellence 2023–2027), University of Milan, 20122 Milan, Italy; 3 Exercise Medicine Unit, Istituto Auxologico Italiano, IRCCS, 20135 Milan, Italy; 4 Department of Medicine, Institute of Sport Medicine and Science, 00197 Rome, Italy

**Keywords:** autonomic nervous system, vagal activity, heart rate variability, left ventricular hypertrophy, athlete’s heart, echocardiography, cardiac imaging, sport cardiology

## Abstract

The “athlete’s heart” represents a physiological condition consequent to long-term adaptation to sport-specific training loads. Cardiac imaging is a pivotal tool in the assessment of athletes. The study of the autonomic nervous system (ANS) using heart rate variability is becoming increasingly popular in sports fields, given its fundamental role in cardiovascular adaptation to exercise. Unfortunately, many barriers limit the clinical use of this methodology. In this observational study of 330 elite athletes, we used a composite index of ANS control (ANSI), designed to overcome pitfalls, following the hypothesis that studying the ANS could help address cardiac adaptation to high-volume training. In athletes subdivided into three groups considering the level (LOW/HIGH) of combined static/dynamic exercise and the presence [+]/absence [−] of left ventricular hypertrophy (LVH), we found that ANSI showed a progressive increase (LOW: 38.2 ± 27.6%; HIGH-LVH [−]: 52.1 ± 27.2%; HIGH-LVH [+]: 64.4 ± 24.9%, J-T test *p* < 0.001), with significant differences between all groups considered (*p* < 0.001). After adjustment and within the HIGH group, ANSI showed the strongest association with LVH and the highest explanatory power among autonomic variables. In conclusion, ANSI was able to differentiate elite athletes characterized by different cardiac remodeling and workloads (as suggested by different sport disciplines), corroborating the hypothesis for a wider use of ANS evaluation in the sports field.

## 1. Introduction

The “athlete’s heart” represents a common physiological condition consequent to long-term adaptation to sport-specific training loads. It involves morphological, functional and electrical changes [1,2,3] contingent on dynamic and static training loads [4,5]. Nevertheless, elite athletes present a normal left ventricular (LV) functionality characterized by normal ejection fraction, normal diastolic function and normal LV filling pressures [3]. Notably, other cardiac adaptations to exercise could be accounted for, such as oxygen uptake [5] or neurocardiac regulation [6,7]. Autonomic adaptations to exercise represent a relatively novel research field, whose importance is demonstrated by the fundamental role of the autonomic nervous system (ANS) in determining both acute [8,9] and long-term [10,11] effects of exercise, which may differ depending on the workload of exercise performed. High exercise training workloads, frequently characterized by maximal anaerobic exercise, in fact, may determine cardiac adaptation and remodeling in athletes, while aerobic exercise may improve cardiometabolic prognosis, also ameliorating the ANS impairment which marks these chronic conditions [9].

While imaging, particularly echocardiographic examination, is nowadays a common, pivotal tool in athletes’ assessment, the study of the ANS has become increasingly popular in the sports field only in recent decades [11,12,13,14,15,16], and heart rate variability (HRV) is the technique most frequently employed [16]. Unfortunately, although highly informative, ANS remains challenging to study due to the complexity of analytical techniques and the difficulty of interpretation, particularly given the variety of methods used to assess it, the large number of autonomic indices derived and the well-known influence of age and sex on cardiac autonomic control [17,18,19]. These factors represent an important barrier to the use of this methodology in real-world settings and to data interpretation.

To overcome these difficulties, given the fundamentally unitary nature of visceral neural regulation [20], we previously introduced an integrated, composite Autonomic Nervous System Index (ANSI) [21,22], a single composite percentile-ranked proxy of autonomic balance, with higher values indicating better autonomic control. This index has proven to be highly informative in previous studies, both in athletes and in untrained subjects or patients [23,24,25].

In this paper, we hypothesize that the study of ANS might be useful in addressing cardiac adaptation to high workload. The main goal of the present study was, then, to verify if a composite index proxy of ANS control might be able to differentiate elite athletes characterized by different exercise training workloads and different cardiac remodeling as suggested by the presence of physiological left ventricular hypertrophy (LVH).

## 2. Materials and Methods

In this cross-sectional observational study, we studied 330 elite athletes (204 males and 126 females), aged 23.9 ± 6.3 years, who took part in the standard pre-participation screening organized and supported by the National Italian Olympic Committee (CONI).

This research was part of an ongoing study on the assessment of the autonomic nervous system in elite athletes and followed the principles of the Declaration of Helsinki and Title 45, US Code of Federal Regulations, Part 46, Protection of Human Subjects, Revised 13 November 2001, effective 13 December 2001 and was cleared by the local Ethical Committee of the University of Milano, n 42/19 dated 23 September 2019. Participants gave their consent, and they acknowledged that they could not be identified via this paper and that the authors had fully anonymized their data. All athletes underwent the mandatory annual pre-participation medical examination, and eligibility for competitive sports participation was confirmed in all cases in accordance with national law. They were free of any relevant disease or conditions characterizing an elevated cardiovascular risk. In particular, athletes presenting life-threatening arrhythmias did not receive clearance to participate in competitions, while athletes presenting supraventricular benign arrhythmias, a condition which does not prohibit the release of the clearance, but might influence autonomic or structural cardiac parameters, were not enrolled in the present study [19].

General anthropometric and clinical characteristics by sex are reported in Appendix A (Table A1). All athletes underwent the following evaluations in the morning (between 09:00 and 12:00), at least 2 h after breakfast.

### 2.1. Clinical Evaluation

Comprehensive medical history, physical and biochemical examination were performed, considering hemodynamic (systolic (SAP) and diastolic (DAP) arterial pressure and heart rate) and anthropometric (weight, height, Body Mass Index (BMI)) parameters. A basal ECG and an ECG stress test were performed, in accordance with national law, to release the clearance to participate in competitions. Moreover, a self-report questionnaire was employed to assess the perceptions of stress, fatigue and somatic symptoms.

### 2.2. Echocardiography

Echocardiographic examination was performed at rest using a high-quality echocardiograph (Epiq 7, Philips Medical Systems, Andover, MA, USA) with a 3–5 MHz probe. Measurements of interventricular septal (IVS) and posterior wall (PW) thicknesses, left ventricular end-diastolic diameter, and left atrial end-systolic diameter were obtained by M-mode echocardiography in accordance with American Society of Echocardiography (ASE) recommendations [26].

The left atrial diameter (LADI) was indexed to body surface area (BSA) in accordance with recommended guidelines [26]. The left ventricular mass (LVMI) was determined using the Devereux formula and subsequently indexed to BSA [26]. Relative wall thickness (RWT) was calculated as twice the posterior wall thickness divided by the left ventricular internal diameter. Left ventricular end-diastolic volume (LVEDVI), end-systolic volume (LVESVI), stroke volume (SVI), and ejection fraction (LVEF) were assessed from apical four- and two-chamber views employing the biplane Simpson’s method and indexed to BSA [26]. Left ventricular diastolic function was evaluated via pulsed-wave Doppler analysis of transmitral flow, measuring early (E) and late (A) diastolic peak velocities in the apical four-chamber view with the sample volume positioned at the tip of the mitral leaflets. Tissue Doppler imaging (TDI) was utilized to assess early diastolic myocardial velocity (e′), obtained at the septal mitral annulus. To account for physiological cardiac adaptation to exercise, particularly left ventricular hypertrophy (LVH), athletes were classified according to European and ASE guideline cut-offs based on LVMI [26]. Specifically, individuals were categorized as LVH absent (LVH [−]: <95 g/m^2^ in females, <115 g/m^2^ in males) or LVH present (LVH [+]: ≥95 g/m^2^ in females, ≥115 g/m^2^ in males). Athletes with concentric left ventricular hypertrophy (according to LVMI and RWT limits [26]) were excluded from the study. This exclusion was applied to focus the analysis on the typical phenotype of physiological exercise-induced cardiac remodeling observed in athletes and to avoid potential diagnostic ambiguity arising from patterns that may overlap with non-physiological hypertrophy.

### 2.3. Autonomic Nervous System Assessment

Our non-invasive approach for evaluating autonomic regulation has recently been summarized [19,21,22,27]. Briefly, an electrocardiogram, non-invasive arterial pressure (measured with a Finometer from TNO, Amsterdam, the Netherlands), and respiratory activity (monitored with a piezoelectric belt from Marazza, Monza, Italy) are recorded on a computer. The beat-by-beat data analysis includes a 5 min resting period followed by a 5 min upright recording, processed offline using HeartScope version 2.0 software [28]. The program can analyze signals in both the time and frequency domains. We used an autoregressive algorithm to automatically compute the power and frequency of spectral components within the bandwidth of interest, discarding components with <5% power, which are treated as noise [28]. The software tool was set to consider components with a center frequency of 0.03–0.14 Hz as low frequency and components within the range 0.15–0.35 Hz as high frequency, recalling that “the HF component is synchronous with the respiration”, using a high coherence between RR variability and respiration as a confirmation. The RR interval (ms) and its variability (RR VAR), evaluated through total power (variance in ms^2^), are standard measures of vagal control. In the frequency domain, autoregressive spectral components are analyzed for the low-frequency (LF, approximately 0.1 Hz) and high-frequency (HF, approximately 0.25 Hz; corresponding to respiration) bands, in ms^2^ and normalized units (nu), representing, respectively, a marker of prevalent sympathetic modulation and a marker of prevalent vagal modulation to the sino-atrial node. We also analyzed bivariate indices from RR interval, blood pressure and respiration to compute a frequency domain index of the cardiac baroreflex (Alpha Index). To simplify the clinical interpretation of multiple HRV variables, we developed a unified autonomic index, the Autonomic Nervous System Index (ANSI) [21,22], serving as a proxy for cardiac autonomic evaluation. As described [21,22], the index was derived as a combination of factor analysis results and a clinically optimized radar plot. By applying factor analysis to a multitude of HRV indices, RR, RR interval variance, and ΔRR LFnu were found to be highly representative of the cardiac autonomic information (considering amplitude and oscillatory code modalities). Accordingly, ANSI was constructed following this procedure: first, the percentile rank (PR) transformations of RR, RR interval variance, and ΔRR LFnu are computed within each age-by-sex class. This way, new variables adjusted for age and sex effects are obtained; second, for each subject, a radar plot is built with the values of these three PR transformed variables and the area of the thus obtained triangle computed; third, the PR transformation is applied to the triangle areas to obtain ANSI as a composite normalized indicator ranging in [0, 100]. ANSI is, by design, free of age and sex bias, expressed as a percentile rank (0–100), and serves as a composite proxy for cardiac autonomic regulation, with higher values indicating better autonomic function [21,22].

### 2.4. Sport Category

To account for differences in training load across sport disciplines, a simple classification based solely on isotonic or isometric components was considered inadequate.

Athletes were therefore divided into two groups (LOW and HIGH intensity) according to the combined level (low or high) of static and dynamic components characterizing each sport discipline, with a major focus on the dynamic component, further simplifying the classification originally proposed by Mitchell et al. [4]. In particular:

- the LOW group (n = 138 athletes) comprised: beach volleyball (n = 1); equestrian (n = 22); foil fencing (n = 2); fencing (n = 2); gymnastics (n = 35); golf (n = 5); volleyball (n = 9); table tennis (n = 5); shooting (n = 26); archery (n = 2); sailing (n = 27); beach tennis (n = 2).
- the HIGH group (n = 192 athletes) comprised: athletics (endurance discipline) (n = 5); biathlon (n = 6); canoeing (n = 21); rowing (n = 39); cycling (n = 6); kayaking (n = 11); swimming (n = 66); basketball (n = 5); handball (n = 17); water polo (n = 4); boxing (n = 6); triathlon (n = 6).

Because a more direct quantification of individual annual training load was unavailable, classification was based on the intrinsic characteristics of the sport disciplines rather than on measured training exposure [4]. The HIGH group was further subdivided based on the presence of cardiac remodeling, specifically according to the absence or presence of left ventricular hypertrophy (LVH), into HIGH-LVH [−] and HIGH-LVH [+], thus defining three levels of cardiovascular adaptation. No athlete in the LOW group presented LVH. We also consider the self-reported percentage of training load (% training load) at the time of the evaluation (100% representing the maximum training volume).

### 2.5. Statistical Analysis

Assuming a moderate between-group effect size for ANSI (Cohen’s d = 0.50), a significance level of α = 0.05 and a statistical power of 80%, a minimum of 64 athletes per group were required [29]. The final study population (n = 330), distributed as LOW (n = 138), HIGH-LVH [−] (n = 101) and HIGH-LVH [+] (n = 91), was therefore considered adequate. For logistic regression analyses, 192 subjects were included in the comparison between HIGH-LVH [−] and HIGH-LVH [+], yielding 91 events. The events-per-variable ratio exceeded the recommended threshold [30].

Data are presented as mean ± standard deviation (SD).

We implemented a step-by-step analytical approach that addresses complementary questions.

First, differences between the two groups (LOW vs. HIGH training) were assessed using the Mann–Whitney U test with Bonferroni correction. Comparisons among the three groups (LOW, HIGH-LVH [−], HIGH-LVH [+]) were assessed using the Mann–Whitney U test for each coupled comparison, the Kruskal–Wallis (K-W Test) test to assess overall differences among the three groups and the Jonckheere–Terpstra test (J-T test) as a trend test. In addition to statistical significance, effect sizes (Cohen’s d) [31,32] were reported to provide a standardized estimate of the magnitude of differences. Categorical variables were compared using the chi-square (χ^2^) test. Associations among autonomic variables were examined using Spearman’s correlation analysis.

Second, the general linear model was used to assess whether these associations remained significant after adjustment for age, sex, perceived stress, fatigue, 4SQ score and workload to evaluate autonomic variables in the three groups, with effect sizes reported as partial eta-squared (η^2^) [31].

Finally, binary logistic regression analyses were performed to evaluate the association between autonomic variables and the presence of left ventricular hypertrophy (LVH) within the high-intensity training group, using HIGH-LVH [+] versus HIGH-LVH [−] as the dependent variable. Separate models were used for each autonomic parameter to avoid collinearity, adjusting for age, sex, training load, 4SQ score, perceived stress and fatigue. Results are reported as odds ratios (OR) with 95% confidence intervals (CIs). Model performance was assessed using Nagelkerke R^2^ [33].

Statistical analyses were performed using a commercial software package (IBM SPSS Statistics, version 30.0, IBM Corp., Armonk, NY, USA).

## 3. Results

### 3.1. Baseline Clinical, Echocardiographic, and Autonomic Variables Between Females and Males

A table in the Appendix A (Table A1) reports baseline data for the entire population, subdivided by sex. As expected, the two groups showed many differences, particularly in anthropometric, hemodynamic, and cardiac structural variables. Males showed higher body weight, height, body surface area (BSA), and Body Mass Index (BMI), as well as higher systolic and diastolic arterial pressure. Lipid profile and fasting glucose were in the normal ranges (Table A1).

Male athletes had higher Left Ventricular Mass Index (LVMI), septal and posterior wall thicknesses (IVS and PW), and relative wall thickness (RWT), as well as larger ventricular volumes (*p* < 0.001). In contrast, female athletes showed a higher left ventricular ejection fraction (LVEF) (*p* = 0.024), but all measurements remained within normal limits in both groups. While there were some significant differences in Stroke Volume Index (SVI) and diastolic function between the groups, all parameters remained within normal limits. The two groups also differed in autonomic measures. Females had lower values of the marker of prevalent sympathetic modulation to the sinoatrial (SA) node (LF (nu)) and higher values of the marker of prevalent vagal modulation to the SA node (HF (nu)), as well as a higher baroreflex sensitivity index (Alpha Index). In contrast, no differences were observed in total power of RR interval variability (RR VAR), a standard measure of vagal control. Heart rate was slightly higher in females. ANSI was similar across the two groups, with no significant differences, as expected given its design.

### 3.2. Clinical, Echocardiographic, and Autonomic Variables

When the analysis was extended to the three levels of cardiovascular adaptation (LOW, HIGH-LVH [−], HIGH-LVH [+]) (Table 1 and Table A2), the proportion of females and males was similar across groups (40.6/59.4%, 36.6/63.4%, and 36.3/63.7%, respectively), with no significant differences (χ^2^(2) = 0.581, *p* = 0.748). Age differed between groups, with athletes in the HIGH-LVH [−] group being younger than those in the LOW and HIGH-LVH [+] groups (*p* = 0.006 and *p* < 0.001, respectively, Table A2). The percentage of self-reported training load (%) showed no significant differences in pairwise comparisons, K-W test and J-T test. Stress levels did not follow a clear pattern across groups, with a difference only between HIGH-LVH [−] and HIGH-LVH [+]. Fatigue varied between groups, but without a consistent trend. No differences were observed for the 4SQ score. LVMI, wall thickness, ventricular volumes, and Stroke Volume Index (SVI) increased across groups (*p* < 0.001), whereas LVEF did not differ among groups.

Regarding autonomic parameters across the three groups, HR decreased progressively from LOW to HIGH-LVH [+] (*p* < 0.001). RR interval variance (RR VAR) together with LF and HF, expressed as an absolute measure, showed a significant increasing trend (*p* < 0.001), although results were variable and did not always reach significance when comparing groups. Normalized indices (LF, HF, LF/HF) and ΔLF showed only partial differences between groups and generally small effect sizes.

In contrast, ANSI showed a progressive increase across groups (LOW: 38.2 ± 27.6%; HIGH-LVH [−]: 52.1 ± 27.2%; HIGH-LVH [+]: 64.4 ± 24.9%, J-T test *p* < 0.001), with significant differences between all groups considered (*p* < 0.001).

Interestingly, the percentage of athletes eventually selected to participate in the 2016 Rio Olympic Games was significantly higher in the HIGH-LVH [+] group than in the HIGH-LVH [−] group, at 40.7% and 16.8%, respectively (χ^2^ = 17.85, *p* < 0.001).

### 3.3. Correlation Analysis of Autonomic Variables

**Figure 1:**
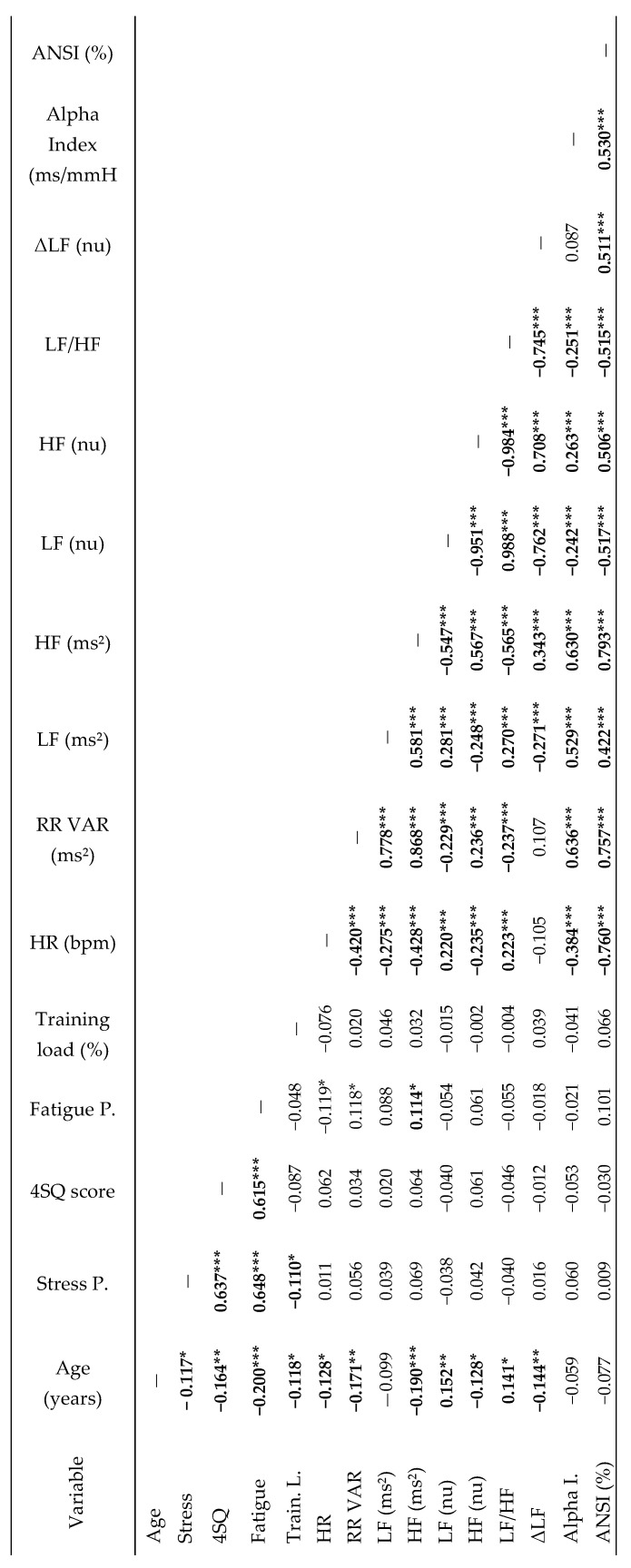
Correlation Matrix. Abbreviations: Age, age (years); Stress, perceived stress score (arbitrary units); Fatigue, perceived fatigue score (arbitrary units); 4SQ, self-report questionnaire score assessing stress, fatigue and somatic symptoms (arbitrary units); P. Training Load, self-reported training load expressed as percentage of maximum training volume at the time of evaluation (%); HR, heart rate (beats per minute); RR VAR, RR interval variance (ms^2^); LF, low-frequency component of RR variability (ms^2^); HF, high-frequency component of RR variability (ms^2^); nu, normalized units; LF/HF, ratio of low- to high-frequency components; ΔLF, stand–rest difference in low-frequency component (nu); Alpha I., index of baroreflex sensitivity expressed in ms/mmHg; ANSI, Autonomic Nervous System Index (%). Data are presented as Spearman’s rank correlation coefficients (ρ). Significant correlations are shown in bold (* *p* < 0.05; ** *p* < 0.01; *** *p* < 0.001).

The correlation matrix (Figure 1) showed that many single autonomic variables were significantly associated with age and sex. Conversely, ANSI was not significantly associated with age and sex, as expected from its design. Of particular note, perceived stress, fatigue, the 4SQ, and training status showed few significant connections with the autonomic variables, suggesting a less relevant influence on autonomic regulation in this cohort.

### 3.4. General Linear Model of Autonomic Variables 

**Figure 2:**
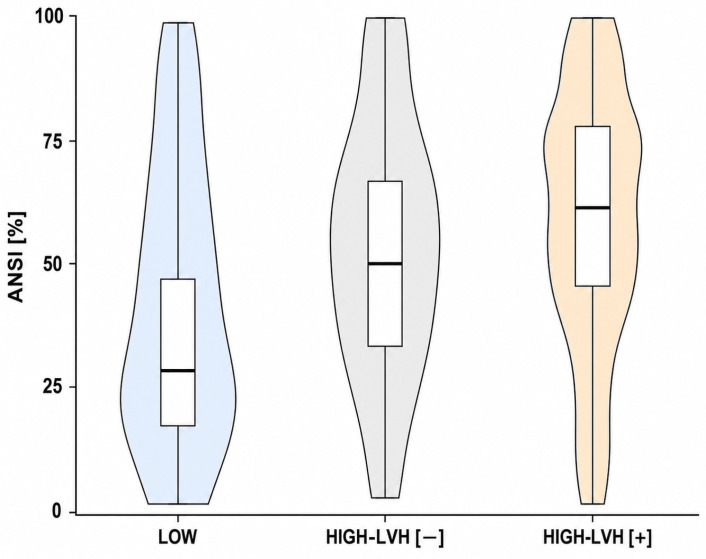
ANSI in the different cardiovascular remodeling groups of elite athletes. Distribution of ANSI values across sport categories. Violin plots show the distribution of ANSI values, with embedded box plots indicating the median and interquartile range. ANSI = Autonomic Nervous System Index (expressed as a percentage, range 0–100); LOW = sport category characterized by a low level of combined static and dynamic exercise components; HIGH-LVH [−] = sport category characterized by a high level of combined static and dynamic exercise components, without left ventricular hypertrophy; HIGH-LVH [+] = sport category characterized by a high level of combined static and dynamic exercise components, with left ventricular hypertrophy. After adjustment for age, sex, 4SQ score, perceived stress, fatigue, and training load, ANSI remained significantly different across all three groups (GLM, *p* < 0.001).

To assess these effects, a general linear model was performed across groups, adjusted for age, sex, perceived stress, fatigue, 4SQ score and training load (Table 2). After adjustment, autonomic variables showed weaker associations with smaller effect sizes. In contrast, ANSI maintained stronger associations with groups (*p* < 0.001). Notably, ANSI showed the highest ability to distinguish between HIGH-LVH [−] and HIGH-LVH [+] (*p* < 0.001), indicating its sensitivity to cardiac remodeling changes (Figure 2).

### 3.5. Logistic Regression Analyses

To further explore the relationship with left ventricular hypertrophy (LVH), logistic regression analyses were performed after excluding the LOW group and considering athletes within the same sport category, allowing comparison between HIGH-LVH [−] and HIGH-LVH [+]. Age, sex, 4SQ score, perceived stress, fatigue, and training load were included as covariates. Among the autonomic variables, ANSI showed the strongest association with LVH (OR 1.022, 95% CI 1.009–1.036, *p* < 0.001) and the highest explanatory power (Nagelkerke R^2^ = 0.214). Heart rate was inversely associated with LVH (OR 0.950, *p* = 0.018, Nagelkerke R^2^ = 0.172), whereas HF (nu) showed a positive association (OR 1.020, *p* = 0.007, Nagelkerke R^2^ = 0.184). LF (nu) (*p* = 0.037) and ΔLF (nu) (*p* = 0.033) showed smaller associations. None of the other autonomic variables, including the Alpha Index, was significantly linked to LVH. Age remained a significant covariate across all models. In contrast, sex, perceived stress, fatigue, 4SQ, and training status did not show independent associations with the outcome.

## 4. Discussion

In this study, we observed that a composite index (ANSI), a proxy of autonomic nervous system control, is associated with a clear and progressive continuum linking the sporting category and cardiac remodeling (expressed by left ventricular hypertrophy, LVH), differentiating athletes from LOW to HIGH-LVH [−] and further to HIGH-LVH [+]. This graduated pattern, consistent across statistical models, was not captured, or only partially captured, by traditional HRV variables, which supports the idea that autonomic adaptation should be viewed as a property of the entire system. These data suggest that, in this cohort of elite athletes, assessment of the autonomic nervous system was associated with sport category and cardiac adaptation, corroborating the hypothesis for a wider use of ANS evaluation in the sports field.

The classification of sport disciplines by their combined dynamic and static components serves as a proxy for chronic hemodynamic load. Over time, this load drives structural cardiac remodeling, characterized by progressive increases in left ventricular mass, wall thickness, and chamber volumes. In this study, we considered elite athletes of the Italian national teams who participated in the selection of the Rio Olympic Games in different disciplines, characterized by different dynamic and static exercise components [4]. In particular, we focus on athletes performing sports of low (such as curling, golf, riflery) and high (such as rowing, cycling, triathlon) levels of combined static/dynamic exercise to differentiate the possible cardiac remodeling best and we considered, among athletes performing high levels of combined static/dynamic exercise, those characterized by the physiological presence [+] of left ventricular hypertrophy (LVH) as compared to those characterized by the its absence [−]. We do not consider athletes characterized by a moderate level (such as synchronized swimming, figure skating, field events) of dynamic and static exercise components (following the Mitchel classification [4]) to differentiate as much as possible the cardiac remodeling of the considered groups.

The observation that athletes in the HIGH-LVH [+] group were significantly older than those in the HIGH-LVH [−] group, together with the independent association of age with the HIGH-LVH [+] group in regression analyses, merits a comment. This data may reflect the chronic greater volume reached in the years of training, which led to increased physiological remodeling, indicating a continuum in the cardiac adaptation to intense training. Consistent with this interpretation, athletes in the HIGH-LVH [+] group also showed a higher prevalence of Olympic selection. This observation is in line with recent evidence from Olympic endurance athletes, in which greater physiological eccentric left ventricular hypertrophy was associated with superior exercise capacity and higher athletic performance [34]. Nevertheless, the present findings should be interpreted only as an association and do not permit causal inferences regarding Olympic selection. Although age contributed to the observed differences between groups, the progressive increase in ANSI remained evident after adjustment for age, suggesting that autonomic adaptation cannot be attributed to age alone. Data of the present study indicate that this structural continuum is mirrored by a similar gradient in autonomic regulation, suggesting that neurocardiac adaptation increases with both the intensity of the sport and the training-induced structural changes in the heart. In this context, the “athlete’s heart” [3] does not represent only a morphological change but a complex phenotype that integrates structure, function, and regulation. The increase in ANSI values (which accounts for age bias) across the various groups supports this interpretation: athletes in higher sports categories (with greater workloads) and those with cardiac remodeling also exhibit better autonomic control, and the combination of these characteristics is associated with a higher ANSI value.

Importantly, despite the progressive increase in LVMI, IVS, PW, LVEDVI, and LVESVI across groups, conventional indices of diastolic function remained preserved. No significant differences were observed for E, E/A, E/e′, or PAPS, while only a modest difference in e′ was detected. However, all values remained within normal ranges, supporting the interpretation that the observed LVH represents a physiological training-induced adaptation rather than a maladaptive process associated with impaired ventricular filling or diastolic dysfunction. Moreover, it should also be considered that all athletes in the study successfully completed the mandatory Italian pre-participation cardiovascular evaluation and were deemed eligible to participate in competitive sport. Consequently, the observed patterns of adaptation are most consistently interpreted as a physiological response.

Therefore, cardiac remodeling and autonomic regulation are considered coordinated responses to long-term training.

ANS evaluation is becoming more and more popular in the sports field and in exercise medicine [11,12,13,14,15,16], due to the important role played by ANS both in acute and chronic cardiovascular adaptation to exercise [8,9,10,11], explaining, albeit in part, the different (even opposite) effects of exercise in determining health and well-being. Acute intense execution of exercise may trigger acute cardiac events, such as arrhythmias or ischemic damage, particularly in athletes with a genetic predisposition [8,9]; conversely, aerobic training may induce a betterment in ANS control, reducing cardiometabolic risk [10], improving well-being [35] and cardio-metabolic prognosis [36]. ANS control plays an essential role in the complex chain of mechanisms responsible for athletic performance, being a fundamental component for successful training in elite athletes. Intermediate levels of training load are associated with signs of increased vagal drive [37,38], whereas, as competition time and training volume intensify, a more complex picture, possibly including sympathetic activation, might be observed [21,37,38]. Moreover, changes in autonomic balance may compromise the quality of training across various loads and possibly signal overreaching and overtraining [39,40].

Spectral analysis of heart rate variability (HRV) is nowadays considered the “de facto” methodology for studying ANS control in clinical settings. It is a simple, non-invasive, cost-effective method that furnishes many variables reflecting the interaction between sympathetic and vagal influences on the heart. Nevertheless, the large-scale use of this non-invasive technique presents some critical methodological and interpretational issues, in particular difficulties in interpreting the large number of variables generated by the analysis and age- and sex-related bias [17,18,19]. To address, albeit in part, the criticalities in HRV interpretation, we introduced the Autonomic Nervous System Index (ANSI) [21,22]. This index integrates information from different autonomic dimensions into a single composite percentile-ranked proxy of autonomic balance, with higher values indicating better autonomic control [21,22]. ANSI is intentionally designed to be free of age and sex bias and demonstrates a strong correlation with cardiac baroreflex gain. It is built by integrating information from highly representative components of the cardiac autonomic system, as indicated by factor analysis, considering a multitude of HRV indices. The 0–100 ranking facilitates simple comparisons among individuals, conditions or time periods and is readily understandable. Notably, ANSI also carries information derived from changes in ANS control during the physiologically induced sympathetic stimulus of active orthostatism [27], reflecting the dynamic nature of cardiac control, which should not be overlooked. ANSI was capable of detecting the effects of aerobic training not only in patients [25] but also in athletes, such as football players and basketball players, and of evidencing the effects of mental relaxation in female football players [23,24].

In previous papers [41], this index was extended to sports, including in its construction parameters derived from recovery after exercise to better represent the dynamics of autonomic regulation in elite athletes and adding more parameters reflecting vagal control [42]. The index provides information on the improvement in autonomic performance with increasing workloads across the Olympic specialties, from archery to cycling [41]. However, the need to perform an exercise stress test may be a barrier to wider adoption of the methodology in real-world sports settings. In a previous study [43], we showed that highly selected elite athletes with physiological cardiac hypertrophy exhibit signs of improved vagal cardiac regulation, particularly markers of vagal recovery after an exercise stress test. In the present study, we add the information that even the “simpler” ANSI (without the need to perform an exercise stress test) was capable of identifying a clear and progressive continuum linking the sporting category and cardiac remodeling, exhibiting moderate to large effect sizes across group comparisons (Cohen’s d ranging from 0.46 to 0.96), supporting the relevance of the observed differences. Notably, in the present cohort, within the HIGH-LVH [+] group, ANSI showed the strongest association with LVH and the highest explanatory power compared with other HRV-derived variables. Importantly, these findings do not imply that other HRV-derived variables are uninformative. Several autonomic indices, including HR, HF (nu), LF (nu), LF/HF ratio, and ΔLF, showed significant differences across groups. However, these differences were generally less consistent across group comparisons and had smaller effect sizes than those observed for ANSI. Therefore, while the single HRV variables provide information on distinct domains of cardiovascular autonomic regulation, ANSI emerged as the most comprehensive and robust indicator of the progressive autonomic adaptation observed across the study groups. Nevertheless, it must be considered as an informative, supportive, clinically interpretable index, not as a standalone discriminator.

Scientific literature on the link between ANS control and physiological cardiac remodeling is limited and yields inconsistent results [44,45], possibly due to the small sample size of elite athletes and differences in protocols and methodologies [19]. For instance, Pluim [44] showed in twelve male cyclists, compared to ten control subjects, that left ventricular mass has no major influence on heart rate variability; Kouidi [45] showed in forty-five athletes as compared to fifteen non-trained subjects that an index built considering some time-domain-derived HRV-derived variables depended on the level of VO_2max_ in endurance-trained athletes but was independent from the extent of myocardial hypertrophy. Vice versa, literature on pathological conditions characterized by LVH, such as hypertrophic cardiomyopathy [46] or hypertension [47], clearly indicates that the LVH significantly correlated with HRV depression, suggesting an autonomic nervous system impairment associated with a pathological form of hypertrophy. Accordingly, the current findings should be regarded as evidence of an association between autonomic regulation, sport category, and physiological LVH in elite athletes. However, causality cannot be inferred from the present cross-sectional study.

Limitations: We have to recognized some limitations of our paper. First, we have no data regarding the molecular response to training that promotes cardiomyocyte proliferation and physiological hypertrophy [3]. Second, we do not have data derived from magnetic resonance, which is considered the gold standard technique to evaluate cardiac morphology and function, for organizational constraints. Although echocardiography represents the standard imaging modality used in athlete evaluation, the absence of cardiac magnetic resonance imaging limits a more comprehensive assessment of myocardial structure. Furthermore, athletes with concentric hypertrophy were excluded to reduce potential diagnostic overlap. Consequently, the present findings should be interpreted as applying primarily to athletes exhibiting the typical pattern of eccentric physiological remodeling. Third, unfortunately, we may not furnish detailed data on the specific weekly training volume or total duration of professional athletic participation. We have to consider that our study population was made up of members of all Italian national teams participating in the selection for the Olympic Games, and that, consequently, they were athletes performing a high training volume. Moreover, we considered all the different types of sport, characterized by different training patterns, rendering it very difficult to have a single parameter capable of reflecting the “real amount” of the training load. We were aware of the importance of having such “parameter” for our research; the solution that we found was to directly ask every athlete the percentage of the training load at the specific moment when we performed our evaluations, considering 100% the maximum training volume (which was different as per different types of sport and single athlete) they were used to performing generally close to the main competition, represented in this specific case by the Olympic Games. This variable reflects the athlete’s current training status and should not be interpreted as an objective measure of annual training exposure. Fourth, the present research is an observational study on a group of 330 elite athletes who perform sports of low or high levels of combined static/dynamic exercise. This design choice aimed to maximize contrast, but it has narrow external validity; consequently, findings apply only to this selected elite-athlete phenotype and should not be generalized to all athletes, recreational exercisers, or the omitted intermediate sport categories, and they need confirmation from a more comprehensive, focused, ideally longitudinal protocol.

## 5. Conclusions

This observational study shows that a simple, composite index, a proxy of autonomic control, is associated with different workloads and cardiac remodeling in elite athletes, corroborating the hypothesis for a wider use of ANS evaluation in the sports field. This finding suggests that athlete’s heart may be characterized by a selective profile of anatomical and neurocardiac remodeling, which can be evaluated by the combination of echocardiography and HRV indices.

## Figures and Tables

**Table 1:** Clinical, echocardiographic, and autonomic variables according to cardiovascular adaptation.

Variables	LOW	HIGH-LVH [−]	HIGH-LVH [+]	K-W Test	J-T Test
	(n. 138)	(n. 101)	(n. 91)		
	Mean ± SD	Mean ± SD	Mean ± SD	Sign.	Sign.
Age (years)	24.75 ± 7.55	21.96 ± 5.28 *	**24.90 ± 4.60 †**	**<0.001**	0.231
Weight (kg)	68.57 ± 13.27	73.17 ± 11.97 *	76.88 ± 13.17 *	**<0.001**	**<0.001**
Height (cm)	174.58 ± 10.09	179.60 ± 10.33 *	180.64 ± 8.99 *	**<0.001**	**<0.001**
BSA (m^2^)	1.82 ± 0.22	1.91 ± 0.20 *	1.96 ± 0.21 *	**<0.001**	**<0.001**
BMI (kg/m^2^)	22.35 ± 2.92	22.55 ± 1.97	23.41 ± 2.56 *	**0.010**	**0.004**
Self-Reported Training Load (%)	66.83 ± 21.26	70.59 ± 19.82	71.76 ± 18.14	0.255	0.128
SAP (mmHg)	115.81 ± 13.18	122.58 ± 12.09 *	124.03 ± 11.76 *	**<0.001**	**<0.001**
DAP (mmHg)	65.60 ± 7.50	65.50 ± 7.59	64.00 ± 7.21	0.158	**0.006**
Stress P. (AU)	2.70 ± 2.66	**3.21 ± 2.75**	**2.21 ± 2.47 †**	**0.026**	0.336
Fatigue P. (AU)	2.88 ± 2.54	3.90 ± 2.91 *	**3.06 ± 2.69 †**	**0.030**	0.308
4SQ score (AU)	22.31 ± 20.78	24.89 ± 24.07	18.68 ± 23.14	**0.038**	0.068
LVMI (g/m^2^)	83.31 ± 14.29	93.66 ± 12.73 *	122.60 ± 17.41 *†	**<0.001**	**<0.001**
IVS (mm)	8.88 ± 1.00	9.55 ± 0.95 *	10.82 ± 1.02 *†	**<0.001**	**<0.001**
PW (mm)	8.68 ± 0.93	9.27 ± 0.85 *	10.48 ± 0.87 *†	**<0.001**	**<0.001**
RWT	0.35 ± 0.02	0.36 ± 0.02 *	0.37 ± 0.02 *†	**<0.001**	**<0.001**
LADI (mm/m^2^)	17.88 ± 1.71	**18.16 ± 1.84**	19.31 ± 2.17 *†	**<0.001**	**<0.001**
LVEDVI (mL/m^2^)	55.72 ± 13.33	62.14 ± 10.84 *	72.48 ± 14.73 *†	**<0.001**	**<0.001**
LVESVI (mL/m^2^)	19.17 ± 5.76	22.02 ± 5.22 *	24.66 ± 6.36 *†	**<0.001**	**<0.001**
LVEF (%)	65.50 ± 5.96	64.56 ± 5.76	65.87 ± 6.09	0.287	0.529
SVI (mL/m^2^)	36.55 ± 9.40	40.12 ± 7.97 *	47.82 ± 11.26 *†	**<0.001**	**<0.001**
E (cm/s)	86.89 ± 15.02	86.65 ± 17.62	83.99 ± 13.38	0.306	0.157
A (cm/s)	50.57 ± 11.01	47.15 ± 9.34	46.29 ± 10.00	0.145	**0.007**
E/A	1.80 ± 0.54	1.90 ± 0.54	1.89 ± 0.47	0.693	0.127
e′ (cm/s)	13.47 ± 2.18	**13.60 ± 2.04**	12.96 ± 1.81 *†	**0.010**	0.051
a′ (cm/s)	7.07 ± 1.94	6.46 ± 1.38 *	6.84 ± 1.43	0.217	0.272
E/e′	6.54 ± 1.18	6.41 ± 1.11	6.52 ± 0.87	0.608	0.732
PAPS (mmHg)	21.70 ± 3.96	21.92 ± 4.27	22.61 ± 3.75	0.327	0.111
TAPSE (mm)	24.09 ± 3.68	24.70 ± 3.94	25.60 ± 3.76 *	**0.002**	**0.008**
HR (bpm)	65.22 ± 9.40	58.70 ± 8.45 *	54.90 ± 7.33 *†	**<0.001**	**<0.001**
RR VAR (ms^2^)	4560.31 ± 4987.76	5371.81 ± 5274.22	5412.49 ± 3905.97 *	**0.003**	**0.001**
LF (ms^2^)	1064.66 ± 1197.10	1405.15 ± 2025.30	1196.28 ± 1041.41	0.104	**0.046**
HF (ms^2^)	1925.60 ± 3108.00	2117.34 ± 2647.14	2207.85 ± 2205.89 *	0.007	**0.002**
LF (nu)	41.99 ± 21.30	40.29 ± 19.24	36.49 ± 17.69	0.137	0.058
HF (nu)	52.19 ± 20.98	53.16 ± 20.08	59.33 ± 19.01 *	**0.028**	**0.018**
LF/HF	1.48 ± 2.47	1.10 ± 1.09	0.91 ± 1.19	0.084	**0.048**
ΔLF (nu)	40.73 ± 19.13	42.42 ± 21.26	46.74 ± 19.34	0.125	0.078
Alpha Index	23.91 ± 10.77	33.10 ± 21.25	28.19 ± 15.01	0.053	0.064
ANSI (%)	38.23 ± 27.63	52.07 ± 27.20 *	64.41 ± 24.87 *†	**<0.001**	**<0.001**

**Abbreviations:** LOW = sport category characterized by a low level of combined static and dynamic exercise components; HIGH-LVH [−] = sport category characterized by a high level of combined static and dynamic exercise components, without left ventricular hypertrophy; HIGH-LVH [+] = sport category characterized by a high level of combined static and dynamic exercise components, with left ventricular hypertrophy; BSA, body surface area; BMI, Body Mass Index; Self-Reported Training Load, self-reported training load expressed as percentage of maximum training volume at the time of evaluation; SAP, systolic arterial pressure; DAP, diastolic arterial pressure; Stress P., perceived stress score; Fatigue P., perceived fatigue score; 4SQ score, self-report questionnaire assessing somatic symptoms; LVMI, Left Ventricular Mass Index; IVS, interventricular septum; PW, posterior wall; RWT, relative wall thickness; LADI, left atrial diameter indexed to BSA; LVEDVI, left ventricular end-diastolic volume indexed to BSA; LVESVI, left ventricular end-systolic volume indexed to BSA; LVEF, left ventricular ejection fraction; SVI, stroke volume indexed to BSA; E, early diastolic transmitral flow velocity; A, late diastolic transmitral flow velocity; E/A, ratio of early to late diastolic filling; e′, early diastolic myocardial velocity; a′, late diastolic myocardial velocity; E/e′, ratio of early transmitral flow to myocardial relaxation velocity; PAPS, pulmonary artery systolic pressure; TAPSE, tricuspid annular plane systolic excursion; HR, heart rate; RR VAR, RR interval variance; LF, low-frequency component of RR variability; HF, high-frequency component of RR variability; nu, normalized units; LF/HF, ratio of low- to high-frequency components; ΔLF, stand–rest difference in low-frequency component (nu); Alpha Index, index of baroreflex sensitivity expressed in ms/mmHg; ANSI, Autonomic Nervous System Index; SD, standard deviation; Sign., *p*-value from Mann–Whitney U test; d; K-W, Kruskal–Wallis test for differences among groups; J-T, Jonckheere–Terpstra test for trend. Data are expressed as mean ± SD. Pairwise Mann–Whitney U values are shown unadjusted. * Significant difference vs. LOW group after Bonferroni correction; † Significant difference vs. HIGH-LVH [−] group after Bonferroni correction. Statistically significant values (*p* < 0.05) are shown in bold. Results of the pairwise comparisons, including *p*-values and effect sizes, are reported in Table A2 of the Appendix A.

**Table 2:** General Linear Model of autonomic variables according to cardiovascular adaptation.

Variables	LOW (n = 138)	HIGH-LVH [−] (n = 101)	HIGH-LVH [+] (n = 91)	LOW vs. HIGH-LVH [−]		LOW vs. HIGH-LVH [+]		HIGH-LVH [−] vs. HIGH-LVH [+]	
	Mean ± SD	Mean ± SD	Mean ± SD	Sign.	Partial η^2^	Sign.	Partial η^2^	Sign.	Partial η^2^
HR (bpm)	65.32 ± 9.44	58.81 ± 8.47	55.45 ± 7.46	**<0.001**	0.083	**<0.001**	0.221	**0.026**	0.028
RR VAR (ms^2^)	4597 ± 5030	5403 ± 5317	5557 ± 3977	0.302	0.006	0.126	0.011	0.883	0.001
LF (ms^2^)	1079 ± 1206	1421 ± 2049	1226 ± 1055	0.182	0.010	0.385	0.004	0.182	0.010
HF (ms^2^)	1930 ± 3135	2109 ± 2661	2308 ± 2280	0.446	0.003	0.299	0.005	0.377	0.004
LF (nu)	42.38 ± 21.28	40.68 ± 19.34	36.10 ± 17.64	0.395	0.004	**0.012**	0.030	**0.021**	0.030
HF (nu)	51.75 ± 20.93	52.91 ± 20.29	60.16 ± 18.59	0.212	0.009	**<0.001**	0.051	**0.003**	0.051
LF/HF	1.50 ± 2.49	1.12 ± 1.10	0.89 ± 1.22	0.604	0.002	**0.032**	0.022	**0.043**	0.023
ΔLF (nu)	40.45 ± 19.24	42.45 ± 21.08	47.38 ± 18.66	0.102	0.015	**0.008**	0.033	**0.025**	0.028
Alpha Index	23.85 ± 10.73	33.43 ± 21.49	29.14 ± 15.44	**0.024**	0.041	**0.010**	0.041	0.204	0.013
ANSI (%)	37.99 ± 27.65	52.15 ± 27.02	64.70 ± 24.86	**<0.001**	0.075	**<0.001**	0.197	**<0.001**	0.065

**Abbreviations:** LOW = sport category characterized by a low level of combined static and dynamic exercise components; HIGH-LVH [−] = sport category characterized by a high level of combined static and dynamic exercise components, without left ventricular hypertrophy; HIGH-LVH [+] = sport category characterized by a high level of combined static and dynamic exercise components, with left ventricular hypertrophy; HR, heart rate; RR VAR, RR interval variance; LF, low-frequency component of RR variability; HF, high-frequency component of RR variability; nu, normalized units; LF/HF, ratio of low- to high-frequency components; ΔLF, stand–rest difference in low-frequency component (nu); Alpha Index, index of baroreflex sensitivity expressed in ms/mmHg; ANSI, Autonomic Nervous System Index; SD, standard deviation; Sign., *p*-values derived from general linear model adjusted for covariates; partial η^2^, partial eta squared. Data are expressed as mean ± SD. Effect sizes [31] were interpreted as small (partial η^2^ ≥ 0.01), moderate (partial η^2^ ≥ 0.06), and large (partial η^2^ ≥ 0.14). Statistically significant values (*p* < 0.05) are shown in bold.

## Data Availability

The dataset used in this study is deposited in the Zenodo repository: https://doi.org/10.5281/zenodo.21066170. Requests to access the dataset should be directed to daniela.lucini@unimi.it.

## Abbreviations

The following abbreviations are used in this manuscript:

A	Late diastolic transmitral flow velocity
a′	Late diastolic myocardial velocity
ANS	Autonomic nervous system
ANSI	Autonomic Nervous System Index
AU	Arbitrary units
BMI	Body Mass Index
BSA	Body surface area
DAP	Diastolic arterial pressure
E	Early diastolic transmitral flow velocity
E/A	Ratio of early to late diastolic filling
E/e′	Ratio of early transmitral flow to myocardial relaxation velocity
e′	Early diastolic myocardial velocity
GLM	General linear model
HF	High-frequency component of RR variability
HR	Heart rate
HRV	Heart rate variability
IVS	Interventricular septum
J-T	Jonckheere–Terpstra test
LADI	Left atrial diameter indexed to BSA
LF	Low-frequency component of RR variability
LVH	Left ventricle hypertrophy
LVEDVI	Left ventricular end-diastolic volume indexed to BSA
LVEF	Left ventricular ejection fraction
LVESVI	Left ventricular end-systolic volume indexed to BSA
LVH	Left ventricular hypertrophy
LVMI	Left Ventricular Mass Index
n	Number
nu	Normalized units
OR	Odds Ratio
PAPS	Pulmonary artery systolic pressure
PW	Posterior wall
RR	RR interval
RR VAR	RR interval variance (total power, ms^2^)
RWT	Relative wall thickness
SAP	Systolic arterial pressure
SD	Standard deviation
SVI	Stroke Volume Index
TAPSE	Tricuspid annular plane systolic excursion
ΔLF	Stand–rest difference in low-frequency component (nu)
4SQ	Short Somatic Symptoms Stress-related Questionnaire

## Appendix A

**Table A1:** Comparison of clinical, echocardiographic, and autonomic variables between females and males.

Variables	Females (n = 126)	Males (n = 204)	Mann–Whitney U Test
	Mean ± SD	Mean ± SD	Sign.
Age (years)	23.49 ± 6.34	24.22 ± 6.28	0.199
Weight (kg)	61.91 ± 8.24	78.67 ± 11.66	**<0.001**
Height (cm)	169.99 ± 6.75	182.60 ± 8.96	**<0.001**
BSA (m^2^)	1.71 ± 0.14	1.99 ± 0.19	**<0.001**
BMI (kg/m^2^)	21.39 ± 2.25	23.52 ± 2.45	**<0.001**
Training load (%)	67.67 ± 18.31	70.39 ± 21.03	0.128
SAP (mmHg)	112.02 ± 10.68	125.18 ± 11.68	**<0.001**
DAP (mmHg)	63.77 ± 6.19	65.97 ± 8.04	**0.040**
FPG (mg/dL)	92.32 ± 10.28	96.82 ± 13.14	**0.005**
Total cholesterol (mg/dL)	179.13 ± 38.45	168.58 ± 33.98	**0.004**
LDL cholesterol (mg/dL)	73.98 ± 17.77	61.85 ± 18.67	**<0.001**
HDL cholesterol (mg/dL)	91.66 ± 27.52	95.08 ± 28.30	0.432
Triglycerides (mg/dL)	64.62 ± 24.53	69.53 ± 34.47	0.115
Stress perception (AU)	3.02 ± 2.64	2.53 ± 2.66	**0.004**
Fatigue perception (AU)	3.48 ± 2.77	3.09 ± 2.69	**0.024**
4SQ score (AU)	26.24 ± 25.14	19.54 ± 20.42	0.984
LVMI (g/m^2^)	84.87 ± 16.15	105.00 ± 21.51	**<0.001**
IVS (mm)	8.76 ± 0.96	10.15 ± 1.14	**<0.001**
PW (mm)	8.55 ± 0.92	9.85 ± 1.00	**<0.001**
RWT	0.35 ± 0.03	0.36 ± 0.02	**<0.001**
LADI (mm/m^2^)	19.09 ± 2.01	18.01 ± 1.86	**<0.001**
LVEDVI (mL/m^2^)	55.43 ± 11.52	67.12 ± 14.14	**<0.001**
LVESVI (mL/m^2^)	18.65 ± 4.82	23.62 ± 5.94	**<0.001**
LVEF (%)	66.25 ± 5.34	64.66 ± 5.94	**0.024**
SVI (mL/m^2^)	37.38 ± 8.78	43.94 ± 10.87	**<0.001**
E (cm/s)	89.45 ± 14.20	84.01 ± 15.92	**0.001**
A (cm/s)	49.18 ± 10.75	46.77 ± 9.85	0.084
E/A	1.91 ± 0.54	1.87 ± 0.53	0.467
e′ (cm/s)	13.46 ± 1.98	13.42 ± 2.10	0.721
a′ (cm/s)	6.19 ± 1.52	6.94 ± 1.61	**<0.001**
E/e′	6.72 ± 1.07	6.31 ± 1.01	**<0.001**
PAPS (mmHg)	21.64 ± 3.98	22.56 ± 3.93	**0.018**
TAPSE (mm)	24.28 ± 3.78	24.69 ± 3.90	0.364
HR (bpm)	62.18 ± 9.73	59.27 ± 9.38	**0.013**
RR VAR (ms^2^)	5108.96 ± 5218.61	5003.34 ± 4555.88	0.721
LF (ms^2^)	1139.62 ± 1758.89	1245.65 ± 1262.00	0.467
HF (ms^2^)	2239.60 ± 2926.54	1952.50 ± 2616.60	0.084
LF (nu)	33.62 ± 18.08	43.86 ± 19.85	**<0.001**
HF (nu)	60.88 ± 19.10	50.48 ± 20.12	**<0.001**
LF/HF	0.83 ± 1.27	1.44 ± 2.07	**<0.001**
ΔLF (nu)	44.96 ± 19.92	41.63 ± 19.92	**0.040**
Alpha Index (ms/mmHg)	30.50 ± 17.41	25.94 ± 14.66	**0.004**
ANSI (%)	46.79 ± 28.25	51.48 ± 29.04	0.161

**Abbreviations:** BSA, body surface area; BMI, Body Mass Index; SAP, systolic arterial pressure; DAP, diastolic arterial pressure; LVMI, Left Ventricular Mass Index; IVS, interventricular septum; PW, posterior wall; RWT, relative wall thickness; LADI, left atrial diameter indexed to BSA; LVEDVI, left ventricular end-diastolic volume indexed to BSA; LVESVI, left ventricular end-systolic volume indexed to BSA; LVEF, left ventricular ejection fraction; SVI, stroke volume indexed to BSA; E, early diastolic transmitral flow velocity; A, late diastolic transmitral flow velocity; E/A, ratio of early to late diastolic filling; e′, early diastolic myocardial velocity; a′, late diastolic myocardial velocity; E/e′, ratio of early transmitral flow to myocardial relaxation velocity; PAPS, pulmonary artery systolic pressure; TAPSE, tricuspid annular plane systolic excursion; HR, heart rate; RR VAR, RR interval variance; LF, low-frequency component of RR variability; HF, high-frequency component of RR variability; nu, normalized units; LF/HF, ratio of low- to high-frequency components; ΔLF, stand–rest difference in low-frequency component (nu); Alpha Index, index of baroreflex sensitivity; ANSI, Autonomic Nervous System Index; SD, standard deviation; Sign., significance value from Mann–Whitney U test. Data are expressed as mean ± SD. Statistically significant values (*p* < 0.05) are shown in bold.

**Table A2:** Significance and Effect size of data reported in Table 1.

Variables	LOW vs. HIGH-LVH [−]		LOW vs. HIGH-LVH [+]		HIGH-LVH [−] vs. HIGH-LVH [+]	
	Sign.	d	Sign.	d	Sign.	d
Age (years)	**0.006 ***	0.362	0.082	0.231	**<0.001 ***	0.692
Weight (kg)	**0.002 ***	0.404	**<0.001 ***	0.608	0.069	0.264
Height (cm)	**<0.001 ***	0.494	**<0.001 ***	0.635	0.397	0.122
BSA (m^2^)	**<0.001 ***	0.441	**<0.001 ***	0.652	0.087	0.247
BMI (kg/m^2^)	0.400	0.110	**0.004 ***	0.381	**0.023**	0.331
Self-Reported Training Load (%)	0.173	0.177	0.157	0.189	0.963	0.006
SAP (mmHg)	**<0.001 ***	0.500	**<0.001 ***	0.596	0.527	0.092
DAP (mmHg)	0.912	0.014	0.090	0.226	0.089	0.247
Stress P. (AU)	0.096	0.217	0.170	0.183	**0.007 ***	0.399
Fatigue P. (AU)	**0.008 ***	0.349	**0.027**	0.296	**0.015 ***	0.357
4SQ score (AU)	0.602	0.167	0.426	0.124	0.119	0.281
LVMI (g/m^2^)	**<0.001 ***	0.766	**<0.001 ***	2.503	**<0.001 ***	2.032
IVS (mm)	**<0.001 ***	0.728	**<0.001 ***	1.944	**<0.001 ***	1.330
PW (mm)	**<0.001 ***	0.658	**<0.001 ***	2.012	**<0.001 ***	1.445
RWT	**0.014 ***	0.319	**<0.001 ***	1.027	**<0.001 ***	0.766
LADI (mm/m^2^)	0.477	0.092	**<0.001 ***	0.637	**<0.001 ***	0.573
LVEDVI (mL/m^2^)	**<0.001 ***	0.505	**<0.001 ***	1.190	**<0.001 ***	0.747
LVESVI (mL/m^2^)	**<0.001 ***	0.488	**<0.001 ***	0.842	**0.002 ***	0.451
LVEF (%)	0.743	0.120	0.419	0.106	0.118	0.227
SVI (mL/m^2^)	**0.006 ***	0.362	**<0.001 ***	1.054	**<0.001 ***	0.765
E (cm/s)	0.517	0.042	0.119	0.207	0.310	0.146
A (cm/s)	**0.028**	0.144	0.054	0.255	0.417	0.118
E/A	0.265	0.084	0.439	0.102	0.878	0.022
e′ (cm/s)	0.632	0.012	**0.008 ***	0.355	**0.006 ***	0.404
a′ (cm/s)	**0.010 ***	0.062	0.234	0.158	0.069	0.264
E/e′	0.694	0.028	0.432	0.140	0.340	0.138
PAPS (mmHg)	0.394	0.110	0.496	0.120	0.119	0.226
TAPSE (mm)	0.059	0.245	**<0.001 ***	0.480	0.094	0.243
HR (bpm)	**<0.001 ***	0.732	**<0.001 ***	1.204	**<0.001 ***	0.521
RR VAR (ms^2^)	**0.025**	0.295	**0.001 ***	0.433	0.334	0.140
LF (ms^2^)	0.091	0.219	0.060	0.249	0.887	0.020
HF (ms^2^)	0.143	0.191	**0.001 ***	0.430	0.130	0.219
LF (nu)	0.589	0.070	0.052	0.258	0.161	0.203
HF (nu)	0.785	0.036	**0.012 ***	0.335	**0.030**	0.315
LF/HF	0.789	0.034	**0.035**	0.280	0.076	0.257
ΔLF (nu)	0.548	0.078	**0.023**	0.303	0.167	0.201
Alpha Index	0.050	0.255	0.088	0.227	0.221	0.177
ANSI (%)	**<0.001 ***	0.698	**<0.001 ***	0.959	**0.002 ***	0.459

**Abbreviations:** LOW = sport category characterized by a low level of combined static and dynamic exercise components; HIGH-LVH [−] = sport category characterized by a high level of combined static and dynamic exercise components, without left ventricular hypertrophy; HIGH-LVH [+] = sport category characterized by a high level of combined static and dynamic exercise components, with left ventricular hypertrophy; BSA, body surface area; BMI, Body Mass Index; Self-Reported Training Load, self-reported training load expressed as percentage of maximum training volume at the time of evaluation; SAP, systolic arterial pressure; DAP, diastolic arterial pressure; Stress P., perceived stress score; Fatigue P., perceived fatigue score; 4SQ score, self-report questionnaire assessing somatic symptoms; LVMI, Left Ventricular Mass Index; IVS, interventricular septum; PW, posterior wall; RWT, relative wall thickness; LADI, left atrial diameter indexed to BSA; LVEDVI, left ventricular end-diastolic volume indexed to BSA; LVESVI, left ventricular end-systolic volume indexed to BSA; LVEF, left ventricular ejection fraction; SVI, stroke volume indexed to BSA; E, early diastolic transmitral flow velocity; A, late diastolic transmitral flow velocity; E/A, ratio of early to late diastolic filling; e′, early diastolic myocardial velocity; a′, late diastolic myocardial velocity; E/e′, ratio of early transmitral flow to myocardial relaxation velocity; PAPS, pulmonary artery systolic pressure; TAPSE, tricuspid annular plane systolic excursion; HR, heart rate; RR VAR, RR interval variance; LF, low-frequency component of RR variability; HF, high-frequency component of RR variability; nu, normalized units; LF/HF, ratio of low- to high-frequency components; ΔLF, stand–rest difference in low-frequency component (nu); Alpha Index, index of baroreflex sensitivity expressed in ms/mmHg; ANSI, Autonomic Nervous System Index; SD, standard deviation; Sign., *p*-value from Mann–Whitney U test; d, Cohen’s d effect size. Effect sizes were interpreted according to Cohen’s thresholds [32] (small ≥ 0.2, medium ≥ 0.5, large ≥ 0.8). Pairwise Mann–Whitney U values are shown unadjusted. Statistically significant values (*p* < 0.05) are shown in bold. * indicates statistical significance after Bonferroni correction.
